# Supplementary material for: Sex-Specific Impact of Fkbp5 on Hippocampal Response to Acute Alcohol Injection: Involvement in Alterations of Metabolism-Related Pathways
Source: Cells. 2023 Dec 31;13(1):89. doi: 10.3390/cells13010089 (PMC10778370; doi:10.3390/cells13010089)
Supplement: Supplementary file 1 [file cells-13-00089-s001.zip › cells-2719168-supplementary.pdf]

cells

MDPI

Article

Sex-Specific Impact of Fkbp5 on Hippocampal Response to Acute Alcohol Injection: Involvement in Alterations of Metabolism-Related Pathways

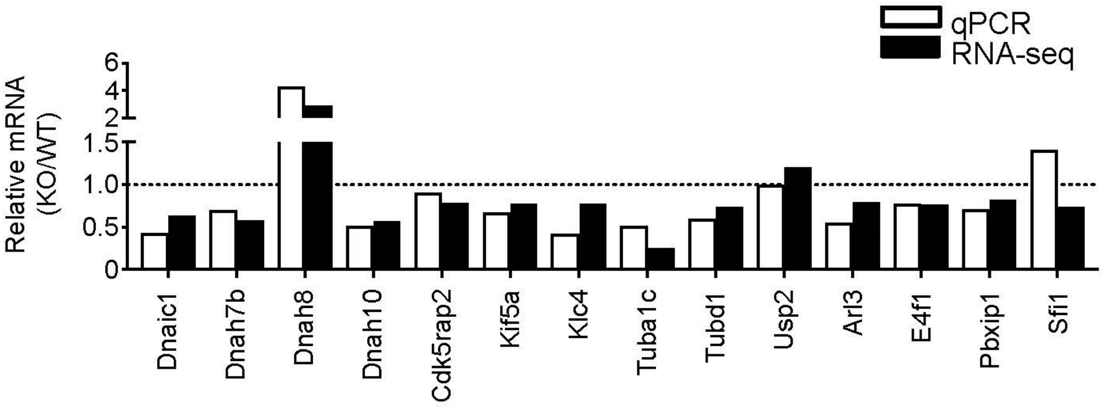

Figure S1. Microtubule-associated gene qRT-PCR confirmation by fold-change between KO and WT.

Naïve network 1-KOWT_adjP05DEG

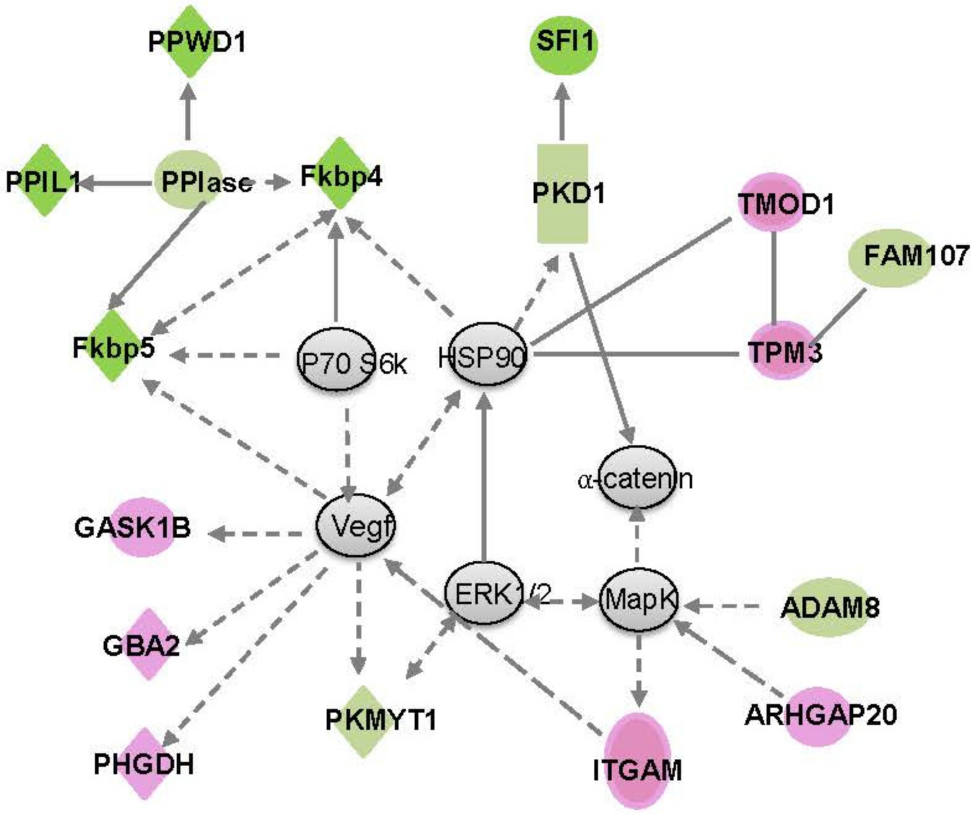

Figure S2. IPA gene network showed *Fkbp5* elimination-associated downregulation of PPIase family members and other genes (colored in green), and up-regulation of some other genes (colored in pink) at baseline.

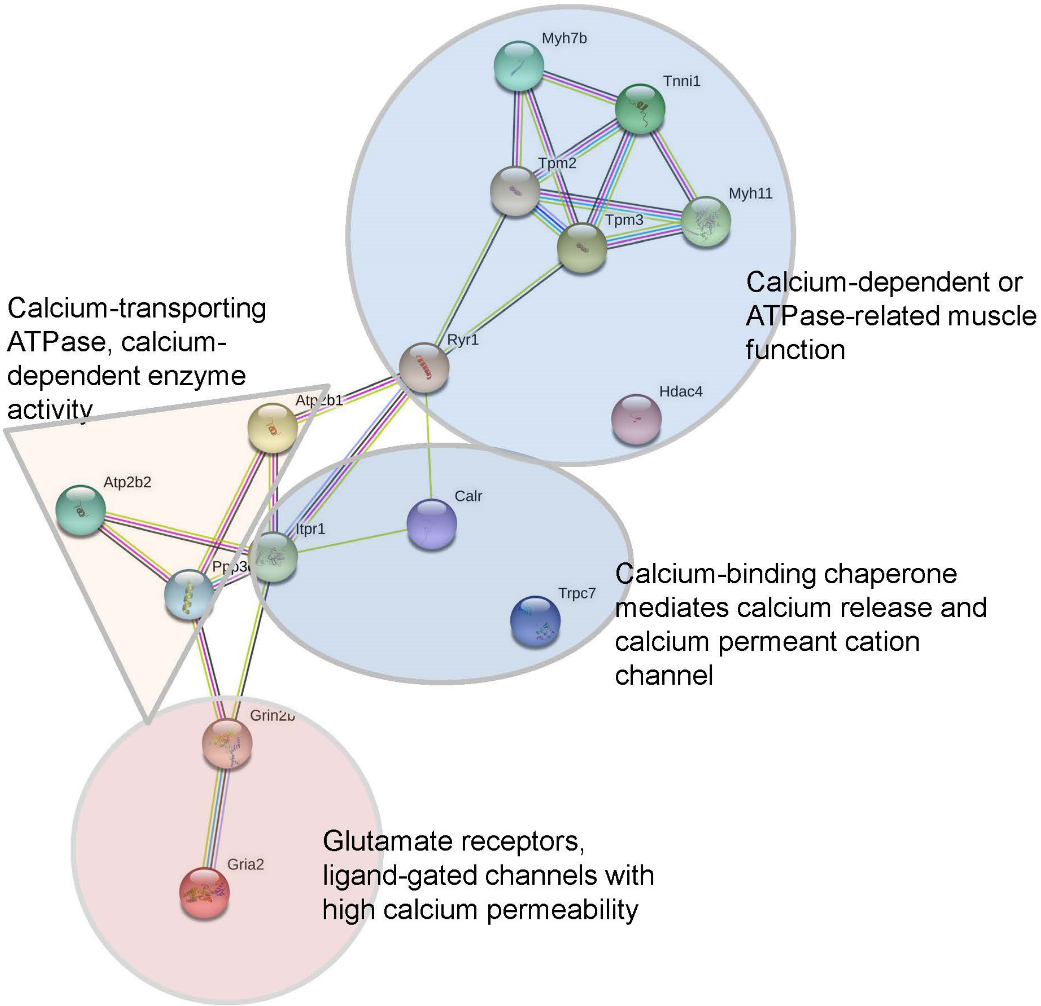

Figure S3. STRING analysis indicated inter-relationship of DEGs that have calcium-dependent, calcium binding, or calcium transporting functions.

Supplemental Tables

Table S1. Disease and functions affected by loss function of *Fkbp5*. The common DEGs (KO vs WT) in both sex were used as input and have identified top disease and function with their significant score and number of focused molecules.

Top Diseases and Functions- Naive	Score	#Focus Molecules
Endocrine System Development and Function, Lipid Metabolism, Small Molecule Biochemistry	47	22
Cell Morphology, Cell-To-Cell Signaling and Interaction, Small Molecule Biochemistry	47	22
Cell Death and Survival, Cellular Compromise, Neurological Disease	31	16
Energy Production, Lipid Metabolism, Small Molecule Biochemistry	26	14
Cell-To-Cell Signaling and Interaction, Cellular Assembly and Organization, Reproductive System Development and Function	17	10

Table S2. Differential expressed gene list with p<0.05, highlighted genes with fold change >2, orange for increasing, and gray for decreasing.

	Naïve Male				Naïve Female				Alc Male				Alc Female			
**gene**	WT	KO	log2FC	padj	WT	KO	log2FC	padj	WT	KO	log2FC	padj	WT	KO	log2FC	padj
**Serpina3m**	1.59	125.89	6.31	7.94E-61	0.84	194.41	7.85	1.29E-23	0.24	152.38	9.31	1.24E-18	1.34	150.96	6.82	6.98E-04
**Serpina3f**	3.24	57.19	4.14	1.73E-20	2.01	101.77	5.66	2.08E-10	2.14	77.53	5.18	4.55E-09	1.52	74.6	5.62	2.94E-02
**Tagap**	39.06	162.26	2.05	2.14E-22	35.4	153.81	2.12	1.04E-21	55.71	168.33	1.6	1.21E-14	57.57	165.24	1.52	1.20E-12
**Tmem181b-ps**	1949.46	7536.09	1.95	6.35E-68	1898.2	6551.39	1.79	4.18E-89	1788	7006.17	1.97	8.34E-43	1929.7	7846.64	2.02	9.53E-98
**Dnahc8**	23.2	86.24	1.89	5.66E-11	18.17	98.66	2.44	1.40E-17	27.21	80.2	1.56	5.84E-07	30.49	82.83	1.44	3.91E-06
**9030025P20Rik**	38.4	128.2	1.74	5.32E-14	37.67	114.64	1.61	4.99E-11	27.45	138.66	2.34	3.44E-22	36.44	144.86	1.99	1.84E-11
**Col2a1**	20.61	67.69	1.72	1.99E-04	25.94	80.03	1.63	1.07E-07	22.68	54.8	1.27	1.46E-03	26.87	71.38	1.41	4.66E-05
**Anxa4**	199.68	511.91	1.36	2.67E-21	172.13	479.54	1.48	7.70E-16	210.5	459.8	1.13	1.80E-06	207.32	548.15	1.4	2.18E-26
**Chia**	34.76	80.19	1.21	2.43E-02	38.86	80.58	1.05	4.29E-03	35.3	94.54	1.42	1.52E-06	43.33	109.54	1.34	6.75E-03
**3110052M02Rik**	532.14	1086.04	1.03	1.18E-24	507.45	1022.21	1.01	3.73E-21	404.7	1009.98	1.32	5.25E-26	414.35	1053.57	1.35	4.39E-34
**Pot1b**	298.93	128.21	-1.22	1.63E-15	268.4	95.34	-1.49	4.13E-21	292.6	107.21	-1.45	2.36E-19	285.92	120.38	-1.25	1.84E-13
**Tagap1**	451.07	171.87	-1.39	4.22E-26	427.14	166.44	-1.36	1.04E-25	399.8	161.05	-1.31	6.80E-20	413.03	166.6	-1.31	4.23E-19
**Folh1**	442.97	167.07	-1.41	3.68E-03	430.57	194.2	-1.15	8.35E-06	410.3	191.85	-1.1	2.09E-03	495.48	238.48	-1.05	7.28E-07
**Abca17**	72.73	16.89	-2.11	1.21E-10	59.89	12.79	-2.23	1.82E-09	68.75	13.15	-2.39	1.87E-12	54.95	19.41	-1.5	2.63E-04
**Tmod4**	77.89	13.25	-2.56	3.32E-15	76.78	13.73	-2.48	2.66E-14	43.14	15.27	-1.5	1.32E-03	42.56	12.79	-1.73	3.92E-04
**Cml5**	72.09	11.93	-2.6	1.99E-14	65.08	12.96	-2.33	7.22E-11	71.28	15.55	-2.2	2.72E-11	49.3	14.14	-1.8	5.46E-05
**Fkbp5**	1653.18	191.71	-3.11	3.42E-124	1670.8	191	-3.13	5.95E-93	2818	326.15	-3.11	2.32E-57	2591.2	271.16	-3.26	9.63E-16
**Pi16**	31.8	66.77	1.07	3.73E-03	24.23	71.28	1.56	1.70E-02	30.9	61.67	1	1.99E-02	34.49	75.7	1.13	2.82E-03
**Glo1**	1665.39	3491.52	1.07	5.35E-37	1689.7	3382.93	1	3.31E-40	1676	3474.84	1.05	7.54E-28	1722.7	3431.18	0.99	7.35E-26
**Marveld2**	104.75	50.17	-1.06	8.21E-05	101.24	49.55	-1.03	2.05E-04	126.1	53.17	-1.25	1.99E-07	119.1	74.11	-0.68	3.21E-02
**Rnps1**	1056.39	499.87	-1.08	3.11E-26	1035.1	501.17	-1.05	2.87E-28	1089	553.9	-0.98	2.15E-18	1138.7	542.36	-1.07	3.78E-22
**Rps2**	1001.07	464.03	-1.11	4.71E-22	986.83	447.14	-1.14	2.56E-32	792.6	424.53	-0.9	7.11E-14	1106	330.07	-1.74	1.52E-54
**Rsph3a**	90.35	38.23	-1.24	1.21E-03	84.96	41.61	-1.03	8.71E-04	85.87	45.12	-0.93	5.28E-03	94.41	36.18	-1.38	4.62E-06
**Pkmyt1**	110.94	45.24	-1.29	3.77E-07	110.42	55.61	-0.99	2.37E-04	117.4	53.96	-1.12	1.80E-05	132.96	55.74	-1.25	5.19E-07
**Tdrd6**	39.9	111.19	1.48	2.46E-09	40.75	94.82	1.22	1.24E-05	41.69	76.77	0.88	1.69E-02	56.34	100.21	0.83	1.04E-02
**Zfp758**	379.52	225.19	-0.75	1.95E-07	429.14	201.71	-1.09	9.42E-16	274.2	153.02	-0.84	9.13E-07	336.48	165.46	-1.02	2.09E-10

**Gpr115**	114.1	59.02	-0.95	3.79E-04	104.43	60.7	-0.78	8.67E-03	109.1	44.83	-1.28	5.60E-07	112.72	46.52	-1.28	2.83E-06
**H2-T24**	249.69	99.59	-1.33	1.81E-15	230.13	109.53	-1.07	4.46E-10	203.3	102.51	-0.99	3.36E-05	247	130.79	-0.92	8.95E-04
**Serpina3n**	1289.06	4005.84	1.64	3.70E-22	1317.4	5617.9	2.09	1.69E-08	1489	4535.35	1.61	6.11E-06	1563.2	4489	1.52	3.34E-01
**Ldb3**	104.4	418.12	2	5.48E-10	143.02	415.78	1.54	1.71E-04	149.4	230.58	0.63	1.00E+00	162.74	434.35	1.42	7.35E-04
**Ccl27a**	170.9	439.47	1.36	1.03E-02	151.71	398.45	1.39	3.03E-03	162.9	499.37	1.62	3.03E-03	315.49	507.54	0.69	9.75E-02
**Tspan11**	55.33	95.69	0.79	6.94E-02	28.35	134.14	2.24	3.61E-07	68.43	146.06	1.09	1.52E-02	49.57	156.08	1.65	1.26E-03
**1500015A07Rik**	524.64	208.03	-1.33	1.09E-26	711.53	196.32	-1.86	2.33E-13	552.6	253.02	-1.13	3.46E-18	502.25	255.94	-0.97	2.47E-01
**Npy2r**	921.68	1667.43	0.86	1.40E-08	845.98	1779.93	1.07	6.25E-08	845.2	1358.35	0.68	7.92E-01	841.57	1332.67	0.66	2.03E-03
**Sh3tc2**	163.2	123.78	-0.4	2.53E-01	261.25	113.96	-1.2	6.13E-03	165.2	85.07	-0.96	1.30E-05	176.21	94.07	-0.91	2.02E-02
**Nudt19**	1697.8	1052.28	-0.69	4.25E-03	1711.2	1055.28	-0.7	1.27E-01	1559	866.33	-0.85	1.63E-07	1713.9	748.28	-1.2	2.14E-31
**Zfp488**	208.17	118.04	-0.82	1.92E-05	195.42	106.1	-0.88	1.90E-02	325.5	243.49	-0.42	4.58E-01	336.63	141.31	-1.25	2.26E-02
**H2-T23**	168.41	83.44	-1.01	9.10E-07	156.57	115.14	-0.44	6.59E-01	221.1	114.06	-0.95	2.60E-04	234.65	126.3	-0.89	3.13E-04
**Eps8l1**	15.37	97.34	2.66	4.87E-01	15.47	168.6	3.45	7.52E-03	13.77	102.22	2.89	9.05E-02	16.47	187.38	3.51	2.80E-02
**Aox4**	33.78	117.1	1.79	7.67E-02	29.17	103.83	1.83	3.95E-12	28.65	102.9	1.84	2.04E-01	25.06	176.28	2.81	8.81E-12
**Pla2g4e**	871.04	344.25	-1.34	6.36E-01	900.89	260.4	-1.79	4.12E-04	794.4	306.3	-1.37	2.63E-01	834.33	300.82	-1.47	8.83E-17
**Rnaset2b**	63.08	17.65	-1.84	8.08E-01	10.82	0.98	-3.47	1.16E-02	132.4	44.39	-1.58	1.00E+00	115.75	0.27	-8.74	2.26E-05
**Gdpd3**	218.09	41.08	-2.41	3.64E-07	162.98	32.29	-2.34	4.34E-27	73.18	36.1	-1.02	1.00E+00	218.53	40.85	-2.42	2.56E-01
**Alpk1**	231.76	481.29	1.05	5.32E-01	222.83	322.9	0.54	8.98E-01	227.2	647.76	1.51	1.01E-05	287.24	1055.27	1.88	4.29E-62
**Vmn2r29**	40.3	51.88	0.36	1.00E+00	29.33	74.34	1.34	1.23E-01	14.79	65.13	2.14	1.40E-02	25.49	85.56	1.75	2.25E-02
**2900052N01Rik**	2753.45	1342.94	-1.04	3.90E-01	2735.9	1312.43	-1.06	2.80E-02	2814	1381.1	-1.03	4.08E-23	2520.9	1589.34	-0.67	2.55E-01
**Slc26a10**	186.91	517.7	1.47	1.05E-04	222.32	565.07	1.35	6.99E-06	360.5	497.47	0.46	1.00E+00	301.31	421.28	0.48	7.57E-01
**4833420G17Rik**	467.52	1120.23	1.26	1.52E-29	465.63	1020.71	1.13	3.51E-01	514.6	907.59	0.82	1.57E-01	535.54	1026.04	0.94	5.63E-11
**Tpm3**	524.03	1173.78	1.16	1.01E-03	525.59	1180.97	1.17	3.28E-04	738.9	1185.78	0.68	3.95E-01	728.91	1182.82	0.7	1.57E-01
**Eif3j**	84.76	180.88	1.09	6.80E-02	72.21	161.32	1.16	1.44E-03	97.27	169.99	0.81	6.17E-02	93.91	157.23	0.74	2.87E-03
**Klk6**	41.83	79.09	0.92	4.80E-01	47.67	111.45	1.23	9.78E-04	36.34	105.69	1.54	1.91E-04	43	73.62	0.78	3.84E-01
**Lyve1**	34.95	43.05	0.3	9.23E-01	21.91	46	1.07	3.82E-02	53.34	81.12	0.6	3.39E-01	35.89	76.63	1.09	1.15E-03
**Nptxr**	147.87	170.3	0.2	9.74E-01	144.52	215.17	0.57	1.79E-01	58.08	160.18	1.46	1.46E-08	365.99	146.77	-1.32	5.35E-09
**Stbd1**	203.09	140.87	-0.53	9.43E-01	197.17	93.93	-1.07	1.60E-02	186.4	140.31	-0.41	1.00E+00	210.34	103.22	-1.03	3.56E-02
**Fxyd2**	111.04	71.72	-0.63	4.71E-01	143.8	71.45	-1.01	4.12E-02	140.6	63.02	-1.16	3.21E-07	142.5	77.81	-0.87	2.61E-01
**Mid1**	1255.6	701.56	-0.84	9.23E-01	3609.4	520.53	-2.79	3.06E-03	566.8	1073.86	0.92	1.00E+00	2592.2	379.77	-2.77	9.30E-03

**Hmgcs2**	519.86	249.89	-1.06	2.53E-04	422.59	312.38	-0.44	5.43E-01	617.5	281.02	-1.14	1.36E-13	522.4	404.62	-0.37	5.97E-01
**Rec8**	172.28	32.65	-2.4	1.51E-29	59.34	48.61	-0.29	1.00E+00	65.24	91.88	0.49	1.00E+00	178.69	49.23	-1.86	1.78E-02
**Pirt**	48.39	99.95	1.05	1.34E-02	51.08	101.53	0.99	3.70E-04	86.93	69.54	-0.32	9.94E-01	60.07	86.28	0.52	7.62E-01
**Casp1**	79.18	143.51	0.86	8.80E-03	78.04	102.24	0.39	8.00E-01	61.21	95.9	0.65	8.66E-01	64.47	153.53	1.25	2.09E-08
**Ddx4**	77.8	139.84	0.85	3.50E-04	75.84	95.28	0.33	6.62E-01	65.89	114.9	0.8	7.77E-01	59.74	128.59	1.11	4.30E-02
**6030422M02Rik**	86.74	110.89	0.35	9.50E-01	81.76	105.08	0.36	9.25E-01	53.57	128.72	1.26	8.13E-03	76.87	143.91	0.9	1.75E-03
**Ccdc80**	153.83	177.57	0.21	9.39E-01	139.49	195.83	0.49	8.40E-02	147.7	235.05	0.67	1.69E-02	144.24	328.35	1.19	5.54E-08
**Pdcd5**	133.82	121.41	-0.14	9.86E-01	137.75	83.69	-0.72	3.98E-02	133.6	91.71	-0.54	1.00E+00	137.28	58.59	-1.23	6.50E-04
**Cdh1**	74.08	62.84	-0.24	1.00E+00	35.41	87.38	1.3	4.41E-03	80.39	42.94	-0.9	1.40E-02	60.96	78.41	0.36	1.00E+00
**Ubc**	3014.73	1816.23	-0.73	7.20E-17	3154.3	2645.71	-0.25	8.35E-01	3451	1631.5	-1.08	5.85E-03	2830.3	2043.2	-0.47	9.34E-02
**Olfml3**	371.38	208.78	-0.83	7.24E-03	310.97	311.31	0	1.00E+00	328.5	205.09	-0.68	1.00E+00	489.22	242.81	-1.01	5.79E-04
**2900041M22Rik**	60.66	29.55	-1.04	1.42E-02	55.75	28.61	-0.96	3.33E-02	41.22	36.94	-0.16	1.00E+00	47.54	28.53	-0.74	3.06E-01
**Gm1821**	287.66	110.51	-1.38	9.83E-01	245.3	24.93	-3.3	9.15E-01	202.9	1749.68	3.11	1.63E-04	1094.5	394.71	-1.47	9.96E-01
**Krt12**	195.58	480.49	1.3	1.60E-01	157.22	393.01	1.32	2.76E-02	381.3	545.09	0.52	1.00E+00	192.27	553.64	1.53	1.86E-01
**Pglyrp1**	57.76	77.37	0.42	9.52E-01	48.79	99.23	1.02	7.11E-02	37.89	133.72	1.82	6.15E-03	70.15	144.18	1.04	3.91E-01
**Rpl28**	68.49	58.22	-0.23	1.00E+00	103.85	41.08	-1.34	1.02E-01	113.7	46.35	-1.29	2.68E-07	110.9	47.85	-1.21	4.27E-01
**Mir5109**	25.13	73.17	1.54	9.97E-01	29.35	18.4	-0.67	8.30E-01	16.83	15.69	-0.1	1.00E+00	120.64	19.09	-2.66	1.75E-03
**Lars2**	1574.47	3891.09	1.31	9.86E-01	2064.5	1321.26	-0.64	5.95E-01	1562	1356.01	-0.2	6.21E-01	6913.1	1476.42	-2.23	1.98E-03
**A930005H10Rik**	290.35	494.96	0.77	6.62E-01	241.79	361.79	0.58	6.46E-01	235	473.39	1.01	5.85E-02	285.89	648.08	1.18	4.59E-21
**Cd180**	32.52	47.3	0.54	5.17E-01	27.15	65.18	1.26	3.69E-02	13.36	27.78	1.06	2.66E-01	23.4	27.18	0.22	1.00E+00
**Ccdc61**	131.04	149.75	0.19	1.00E+00	91.16	188.53	1.05	3.62E-02	74.84	159.4	1.09	8.05E-02	99.88	161.41	0.69	1.61E-01
**Cdh3**	65.52	30.67	-1.1	6.33E-01	60.19	62.88	0.06	1.00E+00	91.15	44.25	-1.04	4.23E-02	79.78	56.12	-0.51	9.28E-01
**Ccrl1**	35.36	13.11	-1.43	4.05E-01	41.42	23.96	-0.79	4.13E-01	84.72	37.57	-1.17	1.19E-04	71.87	38.92	-0.88	6.40E-01
**Trh**	136.54	35.69	-1.94	7.37E-05	70.23	34.27	-1.04	8.89E-01	76.49	62.38	-0.29	1.00E+00	86.72	97.77	0.17	1.00E+00
**Tuba1c**	347.71	89.13	-1.96	1.64E-14	362.76	420.82	0.21	1.00E+00	419.1	193.85	-1.11	8.24E-01	386.86	264.14	-0.55	1.00E+00
**Rad51ap2**	24.44	68.76	1.49	4.95E-03	29.88	48.87	0.71	2.44E-01	41.92	48.04	0.2	1.00E+00	36.02	54.24	0.59	4.24E-01
**Wdfy1**	789.66	1746.75	1.15	1.21E-10	857.37	1501.09	0.81	2.14E-01	955.7	1478.06	0.63	1.89E-01	985.8	1403.06	0.51	3.10E-01
**Cela1**	29.55	62	1.07	2.98E-02	33.69	47.96	0.51	6.20E-01	38.28	57.13	0.58	1.00E+00	40.67	60.8	0.58	7.46E-01
**Myo7a**	629.8	878.1	0.48	7.42E-01	731.9	1056.2	0.53	8.40E-02	894.3	1095.28	0.29	1.00E+00	565.24	1139.06	1.01	8.32E-20
**Iyd**	86.46	120.3	0.48	9.43E-01	49.77	137.59	1.47	1.62E-06	80.95	87.02	0.1	1.00E+00	82.53	81.14	-0.02	1.00E+00

**Anxa9**	70.26	95.46	0.44	5.47E-01	73.97	76.7	0.05	1.00E+00	69.54	98.7	0.51	6.59E-01	70.36	141.03	1	3.66E-05
**Lipg**	53.49	68.66	0.36	6.68E-01	38.88	61.75	0.67	7.22E-01	64.36	90.64	0.49	1.00E+00	45.15	119.54	1.4	2.10E-03
**Slc39a2**	295.99	339.02	0.2	1.00E+00	286.23	269.76	-0.09	1.00E+00	136.8	306.9	1.17	1.27E-04	161.65	269.27	0.74	7.78E-01
**Vill**	233.41	259.92	0.16	1.00E+00	187.72	242.13	0.37	1.00E+00	231.7	283.64	0.29	1.00E+00	165.64	371.54	1.17	1.50E-02
**Snrpf**	91.42	97.73	0.1	1.00E+00	92.21	93.5	0.02	1.00E+00	79.96	93.4	0.22	1.00E+00	141.72	70.37	-1.01	4.40E-05
**Xaf1**	368.71	381.22	0.05	1.00E+00	352.06	364.24	0.05	1.00E+00	125.6	358.23	1.51	1.35E-05	274.44	479.21	0.8	8.19E-01
**Qrfpr**	92.05	91.92	0	1.00E+00	74.95	81.2	0.12	1.00E+00	66.65	92.64	0.47	1.00E+00	54.64	118.92	1.12	2.27E-05
**G530011O06Rik**	192.69	189.52	-0.02	1.00E+00	274.77	277.23	0.01	1.00E+00	150.6	321.44	1.09	6.12E-03	223.96	228.83	0.03	1.00E+00
**F13a1**	86.17	79.59	-0.11	1.00E+00	44.26	100.5	1.18	2.54E-02	71.01	111.89	0.66	5.07E-02	55.52	95.83	0.79	4.34E-01
**Mrc1**	143.05	121.51	-0.24	1.00E+00	66.77	150.29	1.17	4.53E-03	139.7	165.12	0.24	1.00E+00	112.67	173.04	0.62	2.17E-01
**Atp6ap1l**	101.76	83.34	-0.29	1.00E+00	91.46	79.21	-0.21	1.00E+00	99.66	151.31	0.6	1.00E+00	58.2	150.38	1.37	9.80E-04
**Ano2**	151.78	116.07	-0.39	8.98E-01	152.46	70.12	-1.12	1.85E-02	114.7	128.47	0.16	1.00E+00	135.82	119.92	-0.18	1.00E+00
**Cthrc1**	189.74	140.2	-0.44	4.38E-01	189.37	121.93	-0.64	1.64E-01	181.1	130.74	-0.47	6.51E-01	168.36	53.61	-1.65	2.20E-05
**Agxt2l1**	425.61	302.98	-0.49	8.40E-01	308.93	192.3	-0.68	3.00E-01	745.4	477.4	-0.64	2.85E-01	773.98	315.95	-1.29	4.00E-04
**Fcrls**	335.4	236.88	-0.5	9.35E-01	300.72	454.11	0.59	6.96E-01	386.7	239.98	-0.69	1.00E+00	444.78	150.83	-1.56	1.17E-02
**Svep1**	73.06	51.44	-0.51	8.76E-01	75.36	66.05	-0.19	1.00E+00	109.4	48.73	-1.17	1.54E-04	81.88	65.38	-0.32	1.00E+00
**Capza1**	131.65	86.48	-0.61	3.53E-01	120.74	105.11	-0.2	1.00E+00	119.8	84.75	-0.5	1.00E+00	162.57	71.03	-1.19	7.40E-08
**Loxl2**	56.09	29.36	-0.93	4.88E-01	53.6	47.29	-0.18	1.00E+00	96.56	46.84	-1.04	4.21E-02	88.17	46.51	-0.92	3.97E-01
**Exd1**	66.98	34.63	-0.95	1.06E-01	75.72	36.42	-1.06	2.18E-03	59.21	32.59	-0.86	4.10E-01	67.2	41.52	-0.69	1.87E-01
**BC060267**	383.2	190.07	-1.01	3.77E-13	295.69	195.72	-0.6	4.67E-01	256.1	195.15	-0.39	2.05E-01	275.52	226.11	-0.29	6.97E-01
**Ccl9**	62.24	25.51	-1.29	4.37E-04	52.75	35.32	-0.58	8.04E-01	35.11	44.71	0.35	1.00E+00	52.59	45.15	-0.22	1.00E+00

Table S3. DEGs between KO and WT have been identified to play roles in microtubule-associated function.

Category	Term	P-Value	Genes
GOTERM_CC_FAT	GO:0005875~microtubule associated complex	0.01339	KIF5A, DNAHC8, DNAHC7B, KLC4, DNAHC10, DNAIC1
GOTERM_MF_FAT	GO:0003774~motor activity	0.01613	MYO1A, KIF5A, DNAHC8, DNAHC7B, MYH11, KLC4, DNAHC10, DNAIC1
GOTERM_CC_FAT	GO:0030286~dynein complex	0.02472	DNAHC8, DNAHC7B, DNAHC10, DNAIC1
GOTERM_BP_FAT	GO:0007018~microtubule-based movement	0.034836	KIF5A, DNAHC8, DNAHC7B, TUBD1, DNAHC10, TUBA1C
GOTERM_MF_FAT	GO:0003777~microtubule motor activity	0.049777	KIF5A, DNAHC8, DNAHC7B, KLC4, DNAHC10

Table S4. Disease and functions affected by loss function of *Fkbp5*. The common DEGs (KO vs WT) in both sex were used as input and have identified top disease and function with their significant score and number of focused molecules.

Top Diseases and Functions -Alcohol	Score	Focus Molecules
Lipid Metabolism, Small Molecule Biochemistry, Vitamin and Mineral Metabolism	51	22
Cancer, Gastrointestinal Disease, Organismal Injury and Abnormalities	31	15
Cell Death and Survival, Inflammatory Response, Skeletal and Muscular System Development and Function	28	14
Cancer, Cell Morphology, Organismal Injury and Abnormalities	19	10
Cellular Development, Embryonic Development, Organismal Development	14	8

Table S5. A list of primers used for Real-Time PCR quantification.

Gene	*Forward*	*Reverse*
Anxa4	*GTGTGGCATTCGCCGTAGTCTA*	*TCTGGGCATCTTCAGTTGCG*
Arl3	*AACGCGGGCAAGACTACCTT*	*TCTGATTTTCCTCTGCCCGC*
Cc2d1a	*TGGGATTAATGAGGAGGAGCTG*	*ATCCAGGTCTCTCATGCAGAGC*
Ccdc88c	*GCGCATCAACAAGCATGTCA*	*GGGTCTTTGCCAATCATCAAGA*
Decr2	*GGACCATGGAGGAGTGATTGTG*	*TATTCTGGGGACCCCATTCC*
Dnah8	*TGTTTGGGCAAGTATGTGGTTG*	*CCACAGATAAAACGGGCAATTC*
Eps8l1	*CCAAGTGGACAGAGCACCTCAT*	*TCAGCCATAACCACGGTGGA*
Fkbp5	*TGATGAGGGCACCAGTAACAATGG*	*CACCAAACATTGGGGCCTCG*
Folh1	*CTCACACCAGGTTACCCAGCAA*	*TGCTGGACCACCCATGTGTT*
Gapdh	*ATCAACGGGAAGCCCATCAC*	*TTGGCTCCACCCTTCAAGTG*
Gpr111	*GTACAGGAGCCCAGTTGCTGAT*	*TCCACGCAGCAGGTGTTCTT*
GPR115	*GCCATTGTTTTCAGGATCAGCA*	*GCCATTGTTTTCAGGATCAGCA*
Gpr15	*TGTTGCATCACAAGGAGGCT*	*CCGCCACCGCAATAATAACG*
H2-T24	*TGAGGTTTTCACCAATCGGATG*	*ATGTGAACTGGAGGGTGTGAGG*
Igfbp3	*ACACCCAGAACTTCTCCTCCGA*	*AGTTTGGGATGTGGACGCCT*
Khsrp	*ATGGTGGGCTTGATTATTGGC*	*TTTGGACAGACTCAGGTGCTCC*
Kif5a	*GAGCCACAGCATCTTCCTCATC*	*TCTTTGCCTCGTCCAGAACG*
Klk6	*CCCTGCCTTGCTGACTTTCTCT*	*AAGCACAGGGCCAGCATCTT*

Park2	*TTTTCATCTACTGCAAAGGCCC*	*TCACCACTCATCCGGTTTGG*
Phgdh	*GCTCACCTGTGGGATGATCATG*	*TCTGCCGAGGCCAAGAATTC*
Pi16	*ACCTGCGATCCGAACCAGAT*	*GCACACCAGCAAATGGATGTTA*
Pkd1	*GGTGCTGGTGGAACACAATACC*	*ACGTTGGGAAGAACAGTGCG*
Ppil1	*GCCGGAAGTAGTGATTGCTAGC*	*GGGCGCATGCTTCCAGTATA*
PPWD1	*GGTCACCGAGATGCTTGGGTAT*	*GGTCACCGAGATGCTTGGGTAT*
Prss33	*ACCTCCAGATCCTGCTCCTGTT*	*AATGCTTGTTTGCCACGGC*
Rps2	*CCTGTTCTCCCTGCCCATTAAG*	*TCCCCAATAGCGACGAAAGC*
Rpl7	*CAGAGTTGAAGGTGAAGCGCCTG*	*TCCTTGCCATCCTGGCCATC*
Scnm1	*ACTGAGGCTCCTTTGCTAACCC*	*TGCAGTTGGCAAAGGAGGAC*
Serpina3m	*ATGCCAGGCAAGCCAACAA*	*CCCATGTGTCATCTGAGAAGCA*
Sfi1	*GTCTTCGGAGAATGGAGGGAAG*	*TTCCTCATCTCCCGTTGCTG*
Slc4a8	*CTCATCAACGGCTCTGTGCTTC*	*AGGGCTTCCCGCACTTTAAC*
Zfp758	*TGCTACCTGTGTAATCCATGTGT*	*AAATAGCTGGGACCTTCCAGGG*

Table S6. Enriched GO Term in KO and with and without alcohol.

**Naïve-female**				
Annotation Cluster 1	Enrichment Score: 3.7366504535480676			
Category	Term	Count	%	PValue
GOTERM_BP_FAT	GO:0007155~cell adhesion	27	6.733167	7.39E-05
GOTERM_BP_FAT	GO:0022610~biological adhesion	27	6.733167	7.62E-05
GOTERM_BP_FAT	GO:0016337~cell-cell adhesion	15	3.740648	3.27E-04
GOTERM_BP_FAT	GO:0007156~homophilic cell adhesion	10	2.493766	6.14E-04
Annotation Cluster 2	Enrichment Score: 3.197401244427676			
Category	Term	Count	%	PValue
GOTERM_MF_FAT	GO:0043169~cation binding	115	28.6783	1.03E-05
GOTERM_MF_FAT	GO:0043167~ion binding	116	28.92768	1.10E-05
GOTERM_MF_FAT	GO:0046872~metal ion binding	114	28.42893	1.15E-05
GOTERM_MF_FAT	GO:0046914~transition metal ion binding	60	14.96259	0.250968
GOTERM_MF_FAT	GO:0008270~zinc ion binding	48	11.97007	0.316706
Annotation Cluster 3	Enrichment Score: 1.9365110186078869			
Category	Term	Count	%	PValue
GOTERM_CC_FAT	GO:0005788~endoplasmic reticulum lumen	8	1.995012	7.38E-04
GOTERM_CC_FAT	GO:0044432~endoplasmic reticulum part	12	2.992519	0.009251
GOTERM_CC_FAT	GO:0005783~endoplasmic reticulum	22	5.486284	0.227017

Naïve-male				
Annotation Cluster 1	Enrichment Score: 1.9743421279742623			
Category	Term	Count	%	PValue
GOTERM_MF_FAT	GO:0043167~ion binding	99	27.73109	9.05E-04
GOTERM_MF_FAT	GO:0043169~cation binding	95	26.61064	0.003277
GOTERM_MF_FAT	GO:0046872~metal ion binding	91	25.4902	0.011435
GOTERM_MF_FAT	GO:0046914~transition metal ion binding	61	17.08683	0.056392
GOTERM_MF_FAT	GO:0008270~zinc ion binding	50	14.0056	0.070274
Annotation Cluster 2	Enrichment Score: 1.86989562092467			
Category	Term	Count	%	PValue
GOTERM_CC_FAT	GO:0005887~integral to plasma membrane	19	5.322129	0.010102
GOTERM_CC_FAT	GO:0031226~intrinsic to plasma membrane	19	5.322129	0.014549
GOTERM_CC_FAT	GO:0044459~plasma membrane part	43	12.04482	0.016714
Annotation Cluster 3	Enrichment Score: 1.8133129296767974			
Category	Term	Count	%	PValue

GOTERM_CC_FAT	GO:0030016~myofibril	7	1.960784	0.006872
GOTERM_CC_FAT	GO:0043292~contractile fiber	7	1.960784	0.008432
GOTERM_CC_FAT	GO:0030018~Z disc	5	1.40056	0.010243
GOTERM_CC_FAT	GO:0031674~I band	5	1.40056	0.01663
GOTERM_CC_FAT	GO:0044449~contractile fiber part	6	1.680672	0.02178
GOTERM_CC_FAT	GO:0030017~sarcomere	5	1.40056	0.061332

Alcohol-female

Category	Term	Count	%	PValue
Annotation Cluster 1	Enrichment Score: 1.304179881962957			
GOTERM_BP_FAT	GO:0046688~response to copper ion	3	0.955414	0.004397
GOTERM_BP_FAT	GO:0010038~response to metal ion	4	1.273885	0.04408
GOTERM_CC_FAT	GO:0005764~lysosome	7	2.229299	0.066311
GOTERM_CC_FAT	GO:0000323~lytic vacuole	7	2.229299	0.067788
GOTERM_CC_FAT	GO:0005773~vacuole	7	2.229299	0.10932
GOTERM_BP_FAT	GO:0010035~response to inorganic substance	4	1.273885	0.157081
Annotation Cluster 2	Enrichment Score: 1.10397340316513			
GOTERM_MF_FAT	GO:0001871~pattern binding	6	1.910828	0.043345
GOTERM_MF_FAT	GO:0030247~polysaccharide binding	6	1.910828	0.043345
GOTERM_MF_FAT	GO:0030246~carbohydrate binding	10	3.184713	0.049424
GOTERM_MF_FAT	GO:0005539~glycosaminoglycan binding	5	1.592357	0.09196
GOTERM_MF_FAT	GO:0008201~heparin binding	3	0.955414	0.353768
Annotation Cluster 3	Enrichment Score: 1.0317334272219372			
GOTERM_BP_FAT	GO:0006090~pyruvate metabolic process	4	1.273885	0.00585
GOTERM_BP_FAT	GO:0006094~gluconeogenesis	3	0.955414	0.035052
GOTERM_BP_FAT	GO:0034637~cellular carbohydrate biosynthetic process	4	1.273885	0.050522
GOTERM_BP_FAT	GO:0019319~hexose biosynthetic process	3	0.955414	0.052745
GOTERM_BP_FAT	GO:0046364~monosaccharide biosynthetic process	3	0.955414	0.0772
GOTERM_BP_FAT	GO:0046165~alcohol biosynthetic process	3	0.955414	0.104457
GOTERM_BP_FAT	GO:0016051~carbohydrate biosynthetic process	4	1.273885	0.126129
GOTERM_BP_FAT	GO:0006006~glucose metabolic process	4	1.273885	0.345555
GOTERM_BP_FAT	GO:0019318~hexose metabolic process	4	1.273885	0.462015
GOTERM_BP_FAT	GO:0005996~monosaccharide metabolic process	4	1.273885	0.542726

Alcohol-Male

Category	Term	Count	%	PValue
Annotation Cluster 1	Enrichment Score: 1.9322218133369267			
GOTERM_MF_FAT	GO:0003755~peptidyl-prolyl cis-trans isomerase activity	4	2.259887	0.003719
GOTERM_MF_FAT	GO:0016859~cis-trans isomerase activity	4	2.259887	0.004359
GOTERM_BP_FAT	GO:0006457~protein folding	4	2.259887	0.098512
Annotation Cluster 2	Enrichment Score: 1.5911453253393448			
GOTERM_MF_FAT	GO:0043167~ion binding	49	27.68362	0.007785
GOTERM_MF_FAT	GO:0043169~cation binding	48	27.11864	0.010217
GOTERM_MF_FAT	GO:0046872~metal ion binding	47	26.55367	0.014226
GOTERM_MF_FAT	GO:0046914~transition metal ion binding	31	17.51412	0.080096
GOTERM_MF_FAT	GO:0008270~zinc ion binding	25	14.12429	0.122171
Annotation Cluster 3	Enrichment Score: 1.3344374483809975			
GOTERM_CC_FAT	GO:0005604~basement membrane	4	2.259887	0.028302
GOTERM_CC_FAT	GO:0044420~extracellular matrix part	4	2.259887	0.050595
GOTERM_CC_FAT	GO:0005578~proteinaceous extracellular matrix	7	3.954802	0.05243
GOTERM_CC_FAT	GO:0031012~extracellular matrix	7	3.954802	0.061199
